# The diabetic wound microenvironment drives emergence and maintenance of CovRS variants in group B *Streptococcus*

**DOI:** 10.1128/iai.00099-26

**Published:** 2026-06-15

**Authors:** Rebecca A. Keogh, Alyx M. Job, Luke R. Joyce, Madeline S. Akbari, Amber Nguyen, Jeffrey S. Kavanaugh, Alexander R. Horswill, Kelly S. Doran

**Affiliations:** 1 Department of Immunology and Microbiology, University of Colorado Anschutz 129263, Aurora, Colorado, USA; 2 Department of Infectious Diseases and Microbiology, University of Pittsburgh, Pittsburgh, Pennsylvania, USA; 3 Department of Veterans Affairs, Eastern Colorado Healthcare System, Aurora, Colorado, USA; University of Illinois Chicago, Chicago, Illinois, USA

**Keywords:** inflammation, microbial evolution, selection, group B *Streptococcus*, diabetic wounds

## Abstract

Diabetic wounds are often infected with microbes, which perpetuate inflammation, and stall wound healing. The bacterium Group B *Streptococcus* (GBS) is frequently isolated from diabetic wounds; however, little is known about how GBS adapts to survive in this niche. Previously, we found that GBS acquires stable mutations in the major two-component system CovRS during murine diabetic wound infection that result in increased pigmentation. Here, we further characterize these pigmented variants and determine the consequences on GBS survival. Using a murine model of wound infection, we find that *covRS* mutants arise specifically in diabetic hosts and are selected for across multiple GBS backgrounds. Whole genome sequencing of pigmented isolates revealed mutations in both *covR* and *covS*, with most isolates having a single nucleotide insertion or deletion in the *covR* promoter region. Phenotypic analysis of murine-acquired mutants reveals enhanced traits associated with virulence, including increased hemolytic activity, host cell cytotoxicity, and elevated nuclease activity. While our previous and current study indicated that engineered *covR* deletion mutants do not exhibit increased survival in the diabetic wound, we observe that a pigmented isolate survived better than wild-type during co-infection with *Enterococcus faecalis*, another frequently isolated wound pathogen. Finally, we find that depletion of neutrophils reduces the frequency of *covRS* mutant variants that arise in the population. Our work highlights the emergence of *covRS* mutations in GBS, and the consequences of these variations are associated with enhanced virulence and competitive fitness, underscoring the importance of these regulatory changes in the context of diabetic wounds.

## INTRODUCTION

The diabetic wound microenvironment is inflammatory, with a high abundance of phagocytes present. Neutrophils isolated from individuals with diabetes have aberrant functions, including elevated levels of extracellular reactive oxygen species (ROS) production, increased neutrophil extracellular trap (NET) formation, and less phagocytosis of bacteria than neutrophils from non-diabetic individuals (1–4). The consequence is stalled healing, as excess inflammation stalls wound healing and allows for the persistence of numerous bacterial species (5, 6).

*Streptococcus agalactiae*, also known as Group B *Streptococcus* (GBS), is a pathobiont that resides in the gastrointestinal and urogenital tracts of 20%–30% of individuals (7). In immunocompromised individuals, GBS can cause severe infections, including meningitis, pneumonia, and sepsis in neonatal populations, as well as cardiac and skin infections in non-pregnant adults with underlying medical conditions (8–11). GBS infections in non-pregnant adults are on the rise, with diabetes being one of the most common co-morbidities (8, 12, 13). Metagenomic, 16S sequencing, and culture-based techniques have determined that GBS is isolated from diabetic foot ulcers (14–22), with up to 10%–25% of individuals having GBS recovered from wound tissues (13, 17, 19). Interestingly, GBS is absent or rarely isolated from non-diabetic ulcers (13, 23, 24). We previously demonstrated that GBS promotes inflammation in the diabetic wound microenvironment, leading to upregulation and increased production of neutrophil effectors such as myeloperoxidase, elastase, and calprotectin (25, 26). This work also led to the observation that hyper-pigmented bacterial variants, caused by mutations in the two-component system (TCS) *covRS,* arise during GBS diabetic wound infection (25).

CovRS is a TCS comprised of the sensor histidine kinase CovS and response regulator CovR. CovRS is a master regulator, which controls up to 25% of the GBS genome depending on the strain and growth condition tested (27–31). However, most studies agree on a core group of virulence-associated genes which are directly repressed by the response regulator CovR, including the surface hemolysin/cytolysin (hemolytic pigment) encoded by the *cyl* operon, adhesins, such as Srr1 and Srr2, cell-wall-associated factors that bind to host matrix molecules, including FbsA and FbsB, which bind fibrinogen, and the plasminogen-binding protein PbsP (30, 32–34). Consequently, mutations in *covRS* result in the de-repression of these virulence factors, leading to increased GBS-mediated lysis of blood cells and enhanced adhesion to epithelial cells (27, 31, 35). Multiple groups have found that engineered *covR* deletion mutants are hyper-virulent in invasive models of infection, such as sepsis, due to enhanced virulence output (27, 28, 36–38). However, a *covR* deletion mutant is often attenuated in colonization models, such as in vaginal and bladder tissues, which has been attributed to the *covR* mutant triggering an enhanced immune response leading to greater clearance (32, 37, 39). We previously demonstrated that the *cyl* operon and the surface protein PbsP contribute to GBS pathogenesis in diabetic wound infection (25). Due to the acquisition of *covRS* mutations *in vivo*, as well as the importance of two CovR repressed virulence factors in diabetic wound infection, we sought to determine the consequence of these mutations on GBS survival, and the host conditions that promote selection for *covRS* variants within the population.

In this study, we find that the acquisition of *covRS* mutations in the population is specific to a diabetic host and occurs across multiple GBS strain lineages. Phenotypic analysis reveals that mutant murine wound isolates (MWIs) have increased pigmentation, enhanced lysis of host cells including red blood cells, neutrophils, and macrophages, and ability to degrade extracellular DNA in comparison to wild type (WT). Notably, co-infection of WT GBS COH1 or a MWI with *E. faecalis*, revealed that while both GBS strains are highly competitive, the MWI has a significantly higher relative fitness than WT. Our data suggest that *covRS* variants may play a pivotal role in GBS persistence in the polymicrobial wound niche.

## RESULTS

### Mutations in the *covRS* locus are specific to diabetic hosts

**Fig 1:**
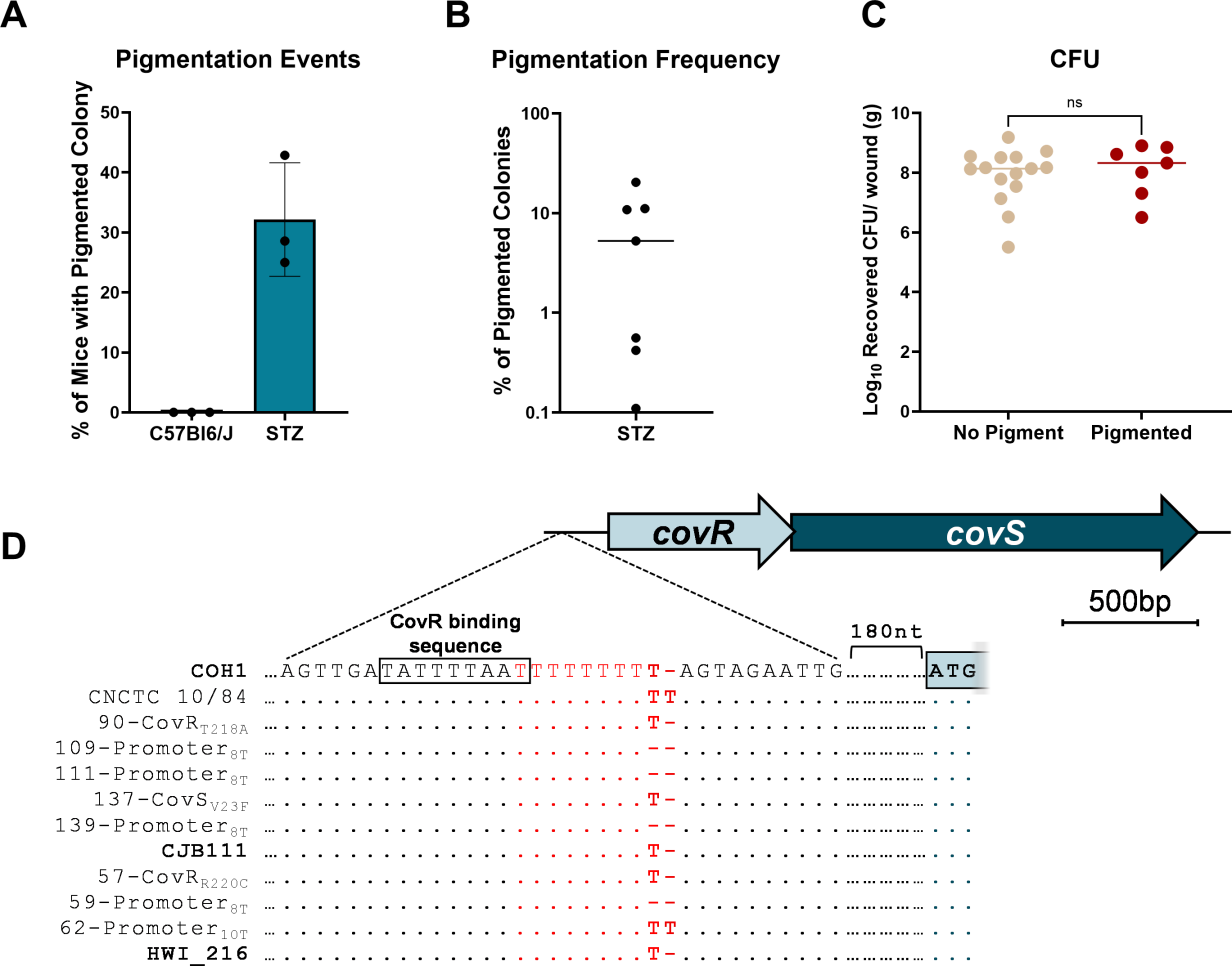
Mutations in the *covRS* locus are specific to diabetic hosts. (**A**) Percentage of mice with pigmented colonies recovered from three separate experiments (pigment/total mice: nDb [0/20], STZ [7/22]). Error bars represent mean and SD. (**B**) Nucleotide alignment of *covR* promoter region with murine wound isolates. (**C**) CFU striated based on whether a pigmentation event occurred (**D**) Nucleotide alignment of covR promoter region with murine wound isolates.

We previously demonstrated that a subset of GBS colonies recovered from murine diabetic wound infection are hyper-pigmented (25). We found that the pigmented murine wound isolates (MWI) in GBS strain A909 contained stable, genetic mutations in the response regulator *covR*, leading to increased pigmentation and hemolysis due to the de-repression of the hemolysin/cytolysin encoded by the *cyl* operon (25). To determine whether the selection for pigmentation events was specific to diabetic animals, we compared the frequency of pigmentation events across three cohorts of non-diabetic (*n* of 6 to 8 per group), as well as three cohorts of streptozotocin (STZ)-induced diabetic mice that were wounded and infected with the GBS strain COH1. Pigmented colonies were recovered from all three cohorts of diabetic mice with 7/22 mice (32%) having pigmented colonies present in wound tissues 4 days post infection (Fig. 1A). Pigmented colonies were recovered in 2/8 mice in experiment 1, 2/7 in experiment 2, and 3/7 in experiment 3 (Fig. 1A). Potential biological differences across experiments could be explained by differences in bacterial concentration and/or immune responses in mice. Conversely, no pigmented colonies were recovered from non-diabetic wounds in any of the three experiments (0/20). The frequency of pigmented colonies varied across the seven mice, with an average of 6.96% of the GBS population exhibiting a pigmented phenotype (Fig. 1B). There was no significant difference in recovered CFU between mice in which a pigmentation event occurred compared with mice where a pigmentation event never occurred (Fig. 1C).

We performed whole-genome sequencing on five colonies, each of which was isolated from an independent mouse in which a pigmented event occurred in COH1. Importantly, colonies were chosen from three independent mouse experiments to minimize effects based on biological differences across experiments. All five encoded either a single nucleotide variant (SNV) in *covR* (GBSCOH1_RS07710) or *covS* (GBSCOH1_RS07705), or a SNP in the *covR* promoter region when compared with the WT COH1 sequence (NCBI Reference Sequence NZ_HG939456), with few additional mutations beyond the *covRS* region (Table 1). Our previous work on pigmented strains in A909 revealed mutations only in the *covR* gene (25), so the hyperpigmented strains reported here are the first identified variants in the *covR* promoter and in *covS* recovered from murine diabetic wound infection. Of note, we recovered fewer bacterial CFU during infection with a *covR* mutant in non-diabetic mice compared with infection with WT (COH1), suggesting that the *covR* mutant is less fit in a healthy host (Fig. S1).

**TABLE 1:** Whole-genome sequencing of murine wound isolates

Isolate	Strain name	Background	Type	Locus tag	Nucleotide change	Amino acid change	Gene
MW_90	90-CovR T218A	COH1	SNV	GBSCOH1_RS07710	652A > G	Thr218Ala	covR
			SNV	GBSCOH1_RS08255	459C > T		Polyprenyl synthetase
			SNV	GBSCOH1_RS03880	10G > T	Glu4^*a*^	
			SNV	Intergenic			
MW_109	109-Promoter8T	COH1	Deletion	191 bp upstream covR			
MW_111	111-Promoter8T	COH1	Deletion	191 bp upstream covR			
MW_137	137-CovS V23F	COH1	SNV	GBSCOH1_RS07705	67G > T	Val23Phe	covS
MW_139	139-Promoter8T	COH1	SNV	GBSCOH1_RS07240	628A > G	Thr210Ala	50S ribosomal protein
			Deletion	191 bp upstream covR			
MW_57	57-CovR R220C	CB111	SNV	ID870_03450	531C > T		Phosphopentomutase
			SNV	ID870_03450	360C > T		Phosphopentomutase
			SNV	ID870_03450	357C > T		Phosphopentomutase
			SNV	Intergenic			
			Deletion	ID870_01600	658C > T	Arg220Cys	covR
			SNV	Intergenic			
MW_59	59-Promoter8T	CB111	SNV	ID870_05060	697A > G	Ile233Val	
			SNV	ID870_03450	531C > T		Phosphopentomutase
			SNV	ID870_03450	360C > T		Phosphopentomutase
			SNV	ID870_03450	357C > T		Phosphopentomutase
			SNV	pepF	411C > A	Phe137Leu	pepF
			SNV	191 bp upstream of covR			
			Deletion	ID870_07110	1336T > C	Ser446Pro	
			SNV	Intergenic			
MW_62	62-Promoter10T	CB111	SNV	ID870_03450	531C > T		Phosphopentomutase
			SNV	ID870_03450	360C > T		Phosphopentomutase
			SNV	ID870_03450	357C > T		Phosphopentomutase
			SNV	191 bp upstream of covR			
			Insertion	Intergenic			

^*a*^ * indicates stop codon.

Interestingly, the SNVs in the promoter region of MWIs 109-Promoter_8T_, 111-Promoter_8T_, and 139-Promoter_8T_ are in the same location as the SNP in the GBS isolate CNCTC 10/84, a hyper-invasive blood isolate of GBS (Fig. 1D). This isolate is known to have low CovRS activity due to a single nucleotide insertion in the *covR* promoter region, resulting in decreased *covR* and *covS* expression and enhanced hemolysis (40). In this paper, Zhu et al. hypothesized that the CNCTC 10T mutation did not alter CovR binding to its promoter but instead affected CovR interaction with RNA polymerase (40). 109-Promoter_8T_, 111-Promoter_8T_, and 139-Promoter_8T_ have a single nucleotide deletion in the same homopolymeric region, and we speculate that these mutations in the promoter region affect CovR interactions with RNA polymerase resulting in decreased transcription of *covRS*.

90-CovR_T218A_ contains a SNV in the CovR coding region leading to a T218A substitution near the C-terminus (Fig. S2A). To determine how this substitution could affect pigmentation, we modeled the amino acid sequence in AlphaFold3 (AF3) (Fig. S2B) (41). Figure S2 shows the AF3-predicted CovR dimer bound to the *cylX* promotor sequence of COH1. The *cylX* gene is the first gene in the operon encoding for the GBS pigment, which is known to be repressed by CovR (34). The ribbon diagram (Fig. S2B) shows only the portion of this sequence that is predicted to directly interact with CovR, which corresponds to the boxed sequence (Fig. S2C). T218A, which is indicated by the yellow star in the top panel and whose side chain is shown as space-filling spheres (carbon in yellow and oxygen in red) in the middle panel, is located on the wing portion, composed of β11 and β12, of the COOH-terminal winged helix-turn-helix DNA-binding domain, where the α8 helix interacts with bases (yellow text in bottom panel) within the major groove, and the wing interacts with bases (green text in the bottom) the preceding minor groove. In the absence of the T218 side chain hydroxyl group, the complex between the T218A mutant and the CovR consensus binding sequence should be less stable since binding would bury the phosphate backbone without an appropriate H-bonding partner, which could account for the increased pigmentation observed for the mutant.

137-CovS_V23F_ is the first pigmented isolate we have identified that contains an amino acid change (V23F) in the sensor histidine kinase CovS (Table 1). Figure S3 shows the AF3-predicted structure for the CovS dimer, with the secondary structure predictions above the color-coded sequence (Fig. S3A) and the ribbon drawing in which each subunit is rainbow colored (blue-to-red corresponds NH_2_ to COOH) (Fig. S3B). The mutation site (indicated by a blue star) is located within the cytoplasmic domain on the α1 helix at the interface between CovS subunits. In the mutant, the F23 side chains from the two subunits pack against one another as well as with I108 and T212 on the α8 helix from the other subunit, which is indicated by the green stars (Fig. S3A). These inter-subunit contacts can influence how the helices in the coiled-coiled region of the CovS dimer pack together and move relative to one another upon activation. In the case of the V23F mutation, disrupting the normal helix might block activation since the mutation lead to increased pigmentation.

Further, we wounded STZ-treated mice as described and infected with the GBS strain CJB111, a serotype V neonatal GBS clinical isolate from a case of late-onset bacteremia (42). Serotype V GBS isolates are emerging in both adults and infants worldwide (43). Pigmented colonies were also recovered from CJB111-infected diabetic mice, and three colonies isolated from independent mice were sent for whole-genome sequencing. All three had SNVs in the *covRS* locus (Table 1), with 57-CovR_R220C_ containing an amino acid substitution R220C in CovR (Fig. S2). R220 is located on the loop connecting β11 and β12 in the wing of the DNA-binding domain and its side chain inserts into the minor groove. The R220 side chain of one subunit occupies a pocket, such that its guanidino group forms hydrogen bonds with all three nucleotides, and the side chain of R220 from the other subunit occupies a similar location and makes hydrogen bonds to several bases. The R220C mutation therefore would eliminate six hydrogen bonds that contribute to binding affinity and binding specificity, so the mutant CovR would be less likely to bind CovR-dependent promoters like the P*cylX*. 59-Promoter_8T_ and 62-Promoter_10T_ contain SNPs in the promoter region, similar to those described above in the COH1 background (Fig. 1D). In addition to mutations in *covRS*, all isolates in the CJB111 background encoded mutations in a putative phosphopentomutase (Table 1). This gene is uncharacterized in GBS but is upstream of *deoD*, a gene involved in the breakdown of nucleosides and deoxynucleosides and thus could be relevant to study.

Finally, we examined a previously published collection of GBS isolates from adults with diabetic wounds from the University of Colorado (13) and found a pigmented isolate from human infection. We performed whole-genome sequencing of the human wound isolate (HWI) 216 and generated a closed genome (Genbank accession: JBTYLZ000000000). Multi-locus sequence typing (MLST) using the PubMLST database identified HWI_216 as sequence type (ST) 335 and clonal complex (CC) 19 (44). Additionally, *in silico* analysis using primers for capsule serotyping revealed HWI_216 is capsule serotype III (45). There were no allelic differences in the *covRS* locus (the 213 nucleotides upstream of *covR*, as in Fig. 1D, and the coding genes ACYPN8_08295 and ACYPN8_08300) when compared with GBS WT strains COH1, CJB111, and A909. We also compared the HWI_216 *covRS* locus to two more similar ST335 CC19 strains, B37VS (NCBI accession number: HG939456) and SG-M426 (NCBI accession number: SAMN05384224) found on NCBI, and again did not identify any allelic differences in the *covRS* locus or promoter region (46, 47). We next examined the *cyl* operon locus in strain HWI_216 as it is responsible for the production of the GBS hemolytic pigment. We compared the region 290 nucleotides upstream of *cylX*, encompassing the promoter and CovR binding sequence (30, 40), through *cylK*, the last gene in the *cyl* operon, between HWI_216 and the five reference strains. HWI_216 contains one allelic variation in *cylD* (ACYPN8_03500) that was not found in any other strain, resulting in a variant at position G99A; however, how this nucleotide variant may be impacting pigment production is not known.

### Hyper-pigmented isolates exhibit enhanced virulence output

**Fig 2:**
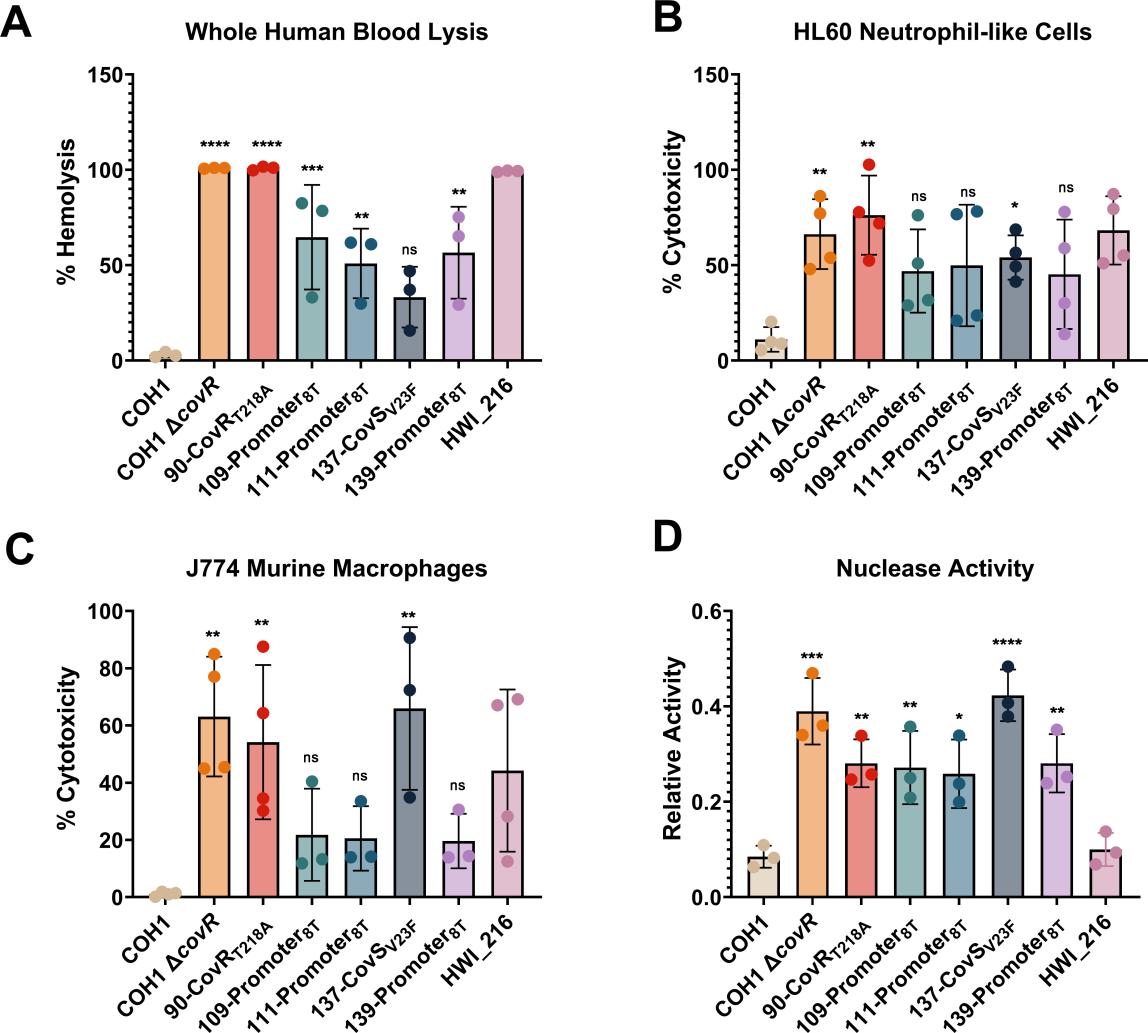
Hyper-pigmented isolates exhibit enhanced virulence output. (**A**) Hemolysis of human red blood cells after 3.5 h incubation. (**B**) Cytotoxicity via LDH release of HL60s of (**C**) J774s after 1.5 h of incubation at MOI of 10. (**D**) Relative nuclease activity. All error bars represent means with SD. Panels A and D performed in biological triplicate. Panels B and C performed in biological quadruplicate. Significance determined via one-way ANOVA with Dunnett’s multiple comparisons against WT as a control group; *P < 0.05, **P < 0.01, ****< 0.0001, ns = not significant.

Upon isolation of hyper-pigmented strains, we sought to characterize the phenotypic consequences of mutations in the *covRS* locus. Pigment production by GBS strains is linked to production of the major cytolysin, encoded by the *cyl* operon, which can lyse red blood cells as well as phagocytes (37, 48–50). Neutrophils, macrophages and red blood cells are some of the most prominent cell types GBS encounters during diabetic wound infection (51). We therefore examined the ability of our MWIs to lyse eukaryotic cells *in vitro*. Whole human blood was isolated from healthy donors and incubated with GBS for 3.5 hours while rotating at 37°C. Incubation of the WT GBS isolate COH1 with whole human blood cells resulted in minimal lysis of red blood cells, however, similar incubation with either a ∆*covR* mutant, or 90-CovR_T218A_, 109-Promoter_8T_, 111-Promoter_8T_, and 139-Promoter_8T_ led to a significant increase in hemolysis (Fig. 2A). 137-CovS_V23F_ had a marked increase in hemolysis albeit not significant. Strains with SNPs in the *covR* promoter region (109-Promoter_8T_, 111-Promoter_8T_, and 139-Promoter_8T_) had an elevated but insignificant difference in lysis of neutrophils or macrophages, suggesting differences in CovR regulation between strains with SNPs in the promoter or the coding sequence. These data are consistent with CovRS mediated repression of the *cyl* operon under WT conditions and de-repression in strains recovered from diabetic wounds (25, 30, 31, 52). HWI_216 had similar hemolytic activity to a ∆*covR* mutant (Fig. 2A). We next measured lactate dehydrogenase (LDH) release as an indicator of eukaryotic cell death and cytotoxicity. Similar trends were observed for GBS-mediated cytotoxicity to human neutrophil-like HL60 cells and murine macrophages (J774s) as pigmented variants exhibited greater cytotoxicity than WT GBS (Fig. 2B and C). Growth of the MWIs in rich media of Todd-Hewitt Broth (THB) demonstrates that each strain has similar growth kinetics to WT, with a minor growth defect in strain 137-CovS_V23F_ (Fig. S4).

Another virulence factor GBS uses to evade the immune system is the extracellular nuclease *nucA* (53). Derre-Bobillot et al. demonstrated that a *nucA* mutant had reduced ability to clear neutrophil extracellular traps and to survive in lung infection (53). It was previously believed that *nucA* regulation was independent of *covRS* (27, 28, 38); however, Mazzuoli et al. demonstrated that *nucA* was under the core regulon of CovRS via transcriptomic analysis and CHIP-seq (30). We therefore sought to address whether pigmented isolates recovered from the diabetic wound had enhanced nuclease activity. To test this, we incubated culture supernatants from strains grown overnight with a single stranded DNA FRET probe (54). We observed that each murine-pigmented isolate had significantly higher nuclease activity than WT, whereas HWI_216 did not exhibit nuclease activity (Fig. 2D). To corroborate our findings, we assessed the virulence outputs of three pigmented strains recovered in the CJB111 background. 57-CovR_R220C_ and 62-Promoter_10T_ exhibited a significant increase in hemolysis, cytotoxicity, and nuclease activity when compared with CJB111 WT (Fig. S5). 59-Promoter_8T_ exhibited a significant increase in hemolysis, nuclease activity, and cytotoxicity toward neutrophil-like cells, however, did not demonstrate a significant increase in cytotoxicity to murine macrophages furthering our hypothesis that strains with SNPs within the promoter region may exhibit different CovR regulation than those with mutations in the coding sequence.

## 90-CovR_T218A_ exhibits increased fitness during polymicrobial diabetic wound infection

**Fig 3:**
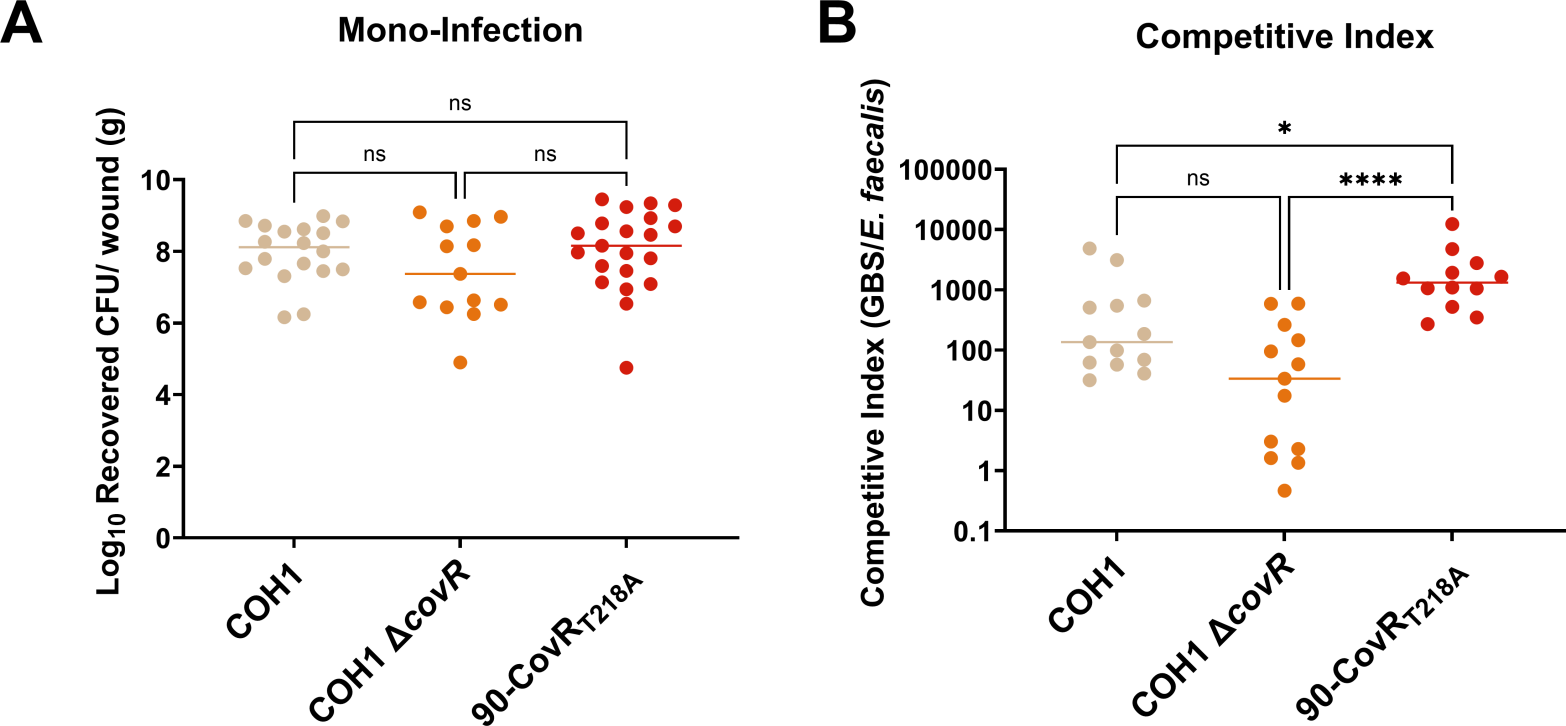
90-COVR_T218A_ exhibits increased fitness during polymicrobial diabetic wound infection. (**A**) GBS CFU recovered from diabetic wound tissue from three independent experiments. (**B**) Competitive index of GBS/ *E. faecalis* in co-infection from two independent experiments. Line at median for both graphs. Significance determined via Kruskal-Wallis with Dunn’s multiple comparisons test; *P < 0.05, ****<0.0001, ns = not significant.

We next determined the consequences of *covRS* mutations *in vivo* in a murine model of diabetic wound infection. We utilized 90-CovR_T218A_ as this strain exhibited comparable virulence output and growth to that of a Δ*covR* mutant (Fig. 2; Fig. S4). STZ-induced diabetic mice were wounded as previously described and infected with either COH1, COH1 Δ*covR,* or 90-CovR_T218A_. After 4 days of infection, we harvested wound tissues to compare bacterial burden. We observed comparable GBS burdens across groups in mono-infection regardless of the GBS input strain (Fig. 3A). This is consistent with our previous findings in murine diabetic wounds where we did not see a difference in bacterial burden with a *covR* mutant in the A909 GBS background (25).

Diabetic wound infections are often polymicrobial in nature, with more than one bacterium present during infection (55–57). Our previous study investigating a collection of bacterial isolates from individuals with diabetic wounds in Colorado indicates that GBS is frequently co-isolated with *E. faecalis* (13). We therefore modeled this by adding *E. faecalis* (13, 56–58) during GBS infection. In the presence of *E. faecalis* strain OG1RF, 90-CovR_T218A_ exhibited a significantly higher competitive index than WT or ∆*covR*, suggesting that 90-CovR_T218A_ is more fit than WT or a Δ*covR* isogenic mutant in polymicrobial infection (Fig. 3B). We also observed that 90-CovR_T218A_ outcompeted *E. faecalis* during growth *in vitro* compared with WT GBS (Fig. S6).

## Neutrophils influence the emergence of hyper-pigmented strains in the diabetic wound microenvironment

Finally, we sought to determine whether specific components of the diabetic wound microenvironment contribute to the acquisition of hyper-pigmented strains *in vivo*. We have previously demonstrated that neutrophils are an abundant immune cell present in GBS-infected diabetic wounds, and that GBS and neutrophils co-localize spatially within diabetic wound tissues (51). Neutrophils produce multiple pro-inflammatory effectors, as well as DNA-damaging agents, such as reactive oxygen and nitrogen species. We therefore hypothesized that neutrophils may contribute to the selection of *covRS* mutants *in vivo*. To test this, we utilized non-diabetic and STZ-induced diabetic mice and performed a wound infection, as previously described (25, 26). Mice were injected with an anti-Ly6G antibody to deplete neutrophils or an isotype (anti-IgG2a antibody) control 1 day prior to infection. We previously demonstrated that addition of anti-Ly6G antibody leads to the ablation of neutrophils in wound tissue on the day of infection (51).

**Fig 4:**
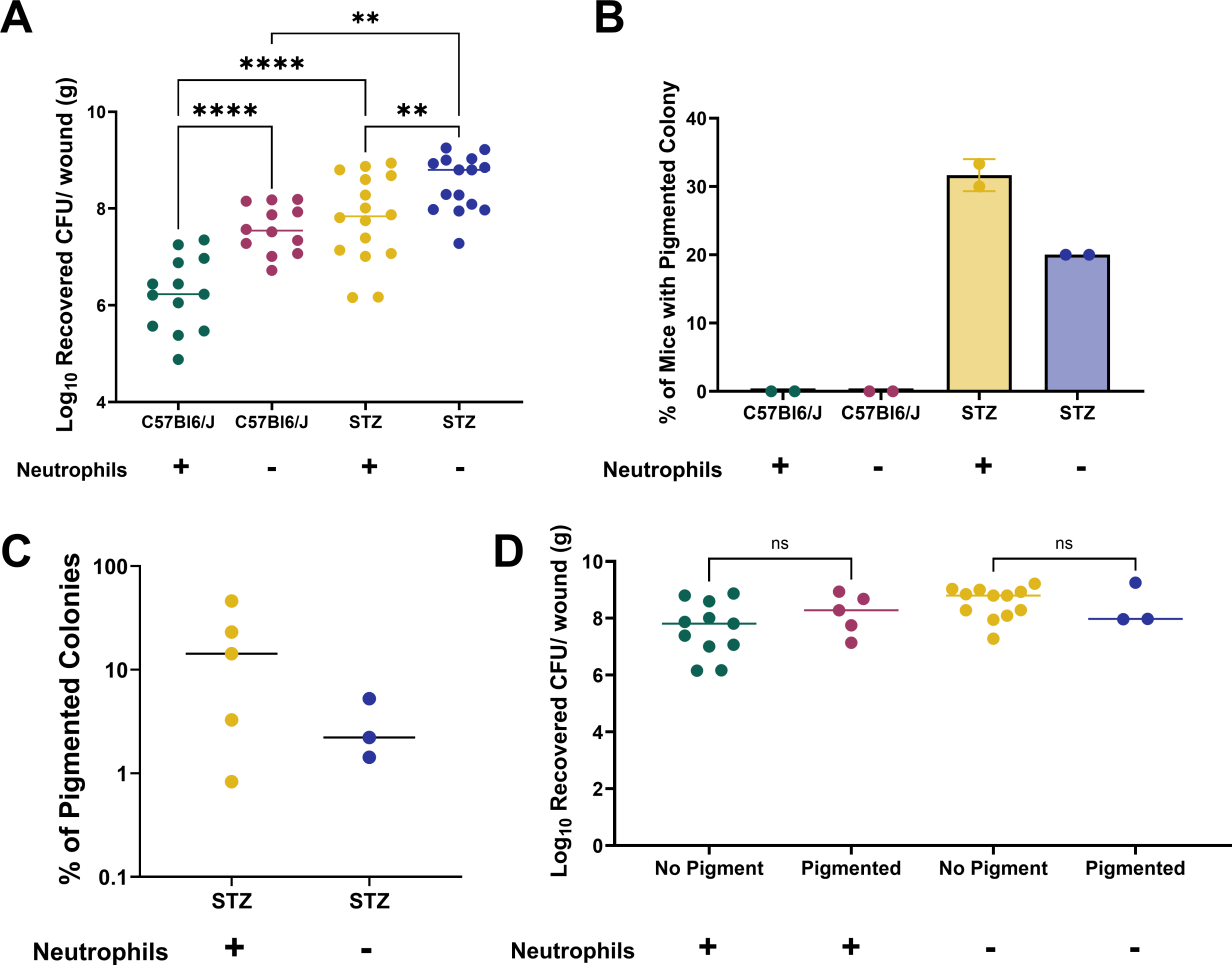
Neutrophils influence the emergence of hyper-pigmented strains in the diabetic wound microenvironment. (**A**) CFU recovered from wound homogenates in two independent experiments (**B**) Percentage of mice with pigmented colonies recovered after neutrophil depletion or control treatment in two independent experiments (pigment/total mice: C57Bl6/J [0/13], C57Bl6/J depleted [0/12], STZ [5/16], STZ depleted [3/15]). (**C**) Pigmentation frequency. (**D**) CFU stratified based on whether a pigmentation event occurred. Significance determined via uncorrected Fisher’s LSD test; *P*-value **<0.01, ****<0.0001.

Depletion of neutrophils led to a significant increase in GBS strain COH1 burden in both C57Bl6/J and STZ-treated mice after 4 days of infection (Fig. 4A). Again, we found that there were no pigmented colonies recovered from C57Bl6/J animals, regardless of the presence or absence of neutrophils (Fig. 4B). Following neutrophil depletion, we observed fewer mice having pigmented colonies recovered after infection in STZ-treated mice (Fig. 4B). The frequency of pigmented colonies within the GBS population also changed, with an average of 17.53% of the population being pigmented in diabetic mice, and only 2.97% of the population being pigmented when neutrophils were depleted (Fig. 4C). There was no difference in CFU between groups when stratified based on whether a pigmentation event occurred (Fig. 4D).

## DISCUSSION

Chronic infections, such as diabetic wounds, impose selective pressure on colonizing microbes, creating opportunities for *in vivo* evolution. It is well documented that microbial populations can evolve within a host via natural selection, and beneficial mutations have been characterized in species, such as *Pseudomonas aeruginosa*, *Streptococcus pneumonia,* and *Salmonella* Typhimurium (59–62). Here, we demonstrate that GBS acquires stable genetic mutations that affect the CovRS TCS during diabetic wound infection. This phenomenon is specific to a diabetic host, as we have not yet recovered a pigmented colony from a wound of a non-diabetic mouse. Work by Shook et al. has defined a similar phenomenon, demonstrating that antibiotic-resistant strains of *S. aureus* emerge and expand in a diabetic host specifically (63). These results suggest that components of the diabetic wound microenvironment specifically may be contributing to this selection.

Within streptococci, the *covRS* locus appears to be an evolutionary hotspot in which mutations frequently arise. In *Streptococcus pyogenes*, multiple studies have identified *covRS* mutations in clinical isolates recovered from humans (64–69). In GBS, Almeida et al. found that disease-specific strains of GBS from the CC17 lineage, which is the most frequent lineage responsible for neonatal infections, had frequent mutations in both *covS* and the serine-threonine kinase *stk1*, a secondary regulator of *covR* (70). Comparative analysis of 923 CC17 strains led authors to determine that mutations affecting the CovRS TCS were the main differentiator between carriage- and disease-associated isolates (70). Pigmented GBS strains have also been identified in hosts with clinical manifestations including prosthetic joint infection and septic arthritis (71). Our group is the first to characterize hyper-pigmented isolates that arise from diabetic wound infection in mice, as well as the first to identify and sequence a hyper-pigmented isolate from human diabetic wound infection.

The importance of *covRS* regulation during GBS pathogenesis has been documented in murine models of meningitis, urinary tract infection, and vaginal colonization (28, 32, 39). Previous studies have shown that the loss of *covRS* leads to de-repression of the *cyl* operon, leading to enhanced hemolysis/cytolysis and resistance to oxidative stress (31, 48, 71). However, most studies utilize clean deletions in *covR* or *covS* to examine the function of this TCS. Of note, Mazzuoli et al. investigated the transcriptome of WT GBS strain BM110, a CC17 strain, using a clean Δ*covR* deletion and a mutant in *covR*::D53A, the site that is phosphorylated by the CovS sensor (30). RNA-seq comparisons of these strains revealed that 79 genes had conserved alterations in transcription when comparing the clean deletion or D53A mutation to WT. However, an additional 71 genes with significant changes in expression were specific to either the clean deletion or D53A only. Authors speculate these differences are likely due to indirect effects of the Δ*covR* deletion or binding of CovR to some promoter regions despite a lack of phosphorylation (30). Our data herein suggest a similar phenomenon, in that murine-acquired isolates with mutations in the *covRS* locus have enhanced pigmentation and hemolysis yet are more fit in polymicrobial infection than an isogenic Δ*covR* mutant.

Many of the isolates identified in this work have SNPs in the *covR* promoter region. Interestingly, while these strains show a significant increase in hemolysis of human red blood cells, they do not exhibit a significant increase in cytotoxicity of neutrophil-like cells or murine macrophages, albeit both are elevated when compared with WT. Work by Zhu et al. identified a SNP in the same region in strain CNCTC 10/84 (40). CNCTC is known to be a hyper-hemolytic strain, and Zhu et al. found that the mutation in the homopolymeric tract led to decreased *covR* and *covS* expression (40). We speculate that the isolates we have recovered with mutations in this region behave similarly and may decrease the expression of *covR* without completely inactivating it the way a complete gene deletion would. Future work determining differences in the regulon between a ∆*covR* mutant, and these MWIs would be necessary to understand whether differences are regulatory in nature, or if additional mutations in the MWI strains contribute to strain-specific differences.

We confirmed that the increased pigmentation in the MWIs corresponded to increased virulence output, including enhanced cytotoxicity toward host cells and hemolysis of human blood. We also found that MWIs exhibit a significant increase in nuclease activity due to the loss of CovR-mediated repression. Diabetes is known to potentiate the formation of neutrophil extracellular traps (NETs) in neutrophils in comparison to non-diabetic controls (1, 72, 73). We speculate that enhanced NucA activity is advantageous in the diabetic wound microenvironment to degrade NETs and escape immune cell clearance.

In this work, we recovered similar burdens of WT and 90-CovR_T218A_ in mono-infection, which is consistent with our previous findings in a different GBS strain background, where we recovered similar CFU during infection with a *covR* deletion mutant compared with WT GBS (25). We hypothesize that a major reason for this is that the WT population is acquiring mutations in the *covRS* locus during infection. Currently, we are unaware of where *covRS* mutations are located spatially within the wound microenvironment. It is therefore possible that a heterogeneous population, with a small subset of *covRS* mutations exhibiting enhanced virulence output and the majority of the population retaining the WT genotype, is advantageous to survival of the whole. Phenotypic and genotypic heterogeneities have been proposed in multiple species, such as *Salmonella typhimurium*, *Pseudomonas aeruginosa*, and *Streptococcus pneumoniae*, as methods for population survival (59, 74–76). Further work on the genotypic and phenotypic heterogeneity of GBS during diabetic wound infection would be beneficial to determine the population dynamics within the diabetic wound microenvironment. We do find that 90-CovR_T218A_ has enhanced relative fitness compared with WT when co-infected with the common wound bacterium *E. faecalis*. We therefore speculate that mutations in the *covRS* locus may be advantageous to retain in the population for when other microbes enter this niche. Further work is necessary to determine whether this competitive advantage is specific to *E. faecalis* or will occur in the presence of additional bacteria present in the diabetic wound.

Another possibility is that alternative mutations in strain 90-CovR_T218A_ confer a fitness advantage in polymicrobial infection. 90-CovR_T218A_ contains three additional mutations based on whole-genome sequencing (Table 1). Of these, there is a silent mutation in GBSCOH1_RS08255 and a mutation in an intergenic region. There is also a truncation in GBSCOH1_RS03880, a gene encoding a putative ABC transporter. This transporter is downregulated during vaginal colonization (77), and the gene encoding the transporter is directly regulated by CovRS (30). We do not know currently if the differences in polymicrobial burden are due to differences in CovRS regulation between the Δ*covR* mutant or the point mutation, due to secondary loss of this ABC transporter, or both.

Finally, we sought to investigate whether the host was contributing to the selection of pigmented strains in the diabetic wound. Previous work by our group has identified that neutrophils are the most abundant cells GBS encounters during early wound establishment, and that GBS and neutrophils often co-localize in the murine model of diabetic wound infection (51). To determine whether neutrophils contributed to mutation or selection for hyper-pigmented strains, we depleted neutrophils in both a healthy and a diabetic host. Depletion of neutrophils led to a significant increase in bacterial recovery in both host backgrounds. Interestingly, despite a 2-log increase in CFU after neutrophil depletion in a healthy host, we still never isolated a single pigmented colony. It is known that neutrophils from individuals with diabetes have aberrant functions in comparison to non-diabetic neutrophils, including increased NET formation, decreased phagocytosis of bacteria, increased extracellular ROS production, and decreased intracellular ROS production (1). We hypothesize that aberrant neutrophil function, including enhanced NET formation, leads to increased mutation on the GBS population *in vivo*. An alternative hypothesis is that mutations are arising in both diabetic and healthy mice, but that neutrophils from healthy individuals can clear *covRS* mutants that arise during infection. Although depletion of neutrophils reduced selection for pigmented colonies, we still found pigmented variants in diabetic mice in the absence of neutrophils. Our data therefore suggests that other factors in the diabetic wound environment, such as other immune cells, increased production of anti-microbial peptides, excess glucose, or other wound pathogens, may contribute to GBS adaptation; however, this is currently unknown and the subject of our future studies.

## MATERIALS AND METHODS

### Bacterial strains and growth conditions

All strains used in the study are listed in Table S1. GBS strains were grown at 37°C statically in Todd-Hewitt broth (THB). Human wound isolate HWI_216 was identified from a previously published collection of GBS clinical isolates from adults with diabetic wounds (13).

### Murine model of wound infection

Eight- to 12-week-old male mice from the C57Bl/6J background were used throughout the study. In groups designated as diabetic, 2 weeks prior to infection, mice were given low-dose injections of streptozotocin (50 mg/kg) (CAS-No: 18883-66-4) dissolved immediately before injection in 100 µL 50 mM sodium citrate buffer (pH 4.5) for five consecutive days, followed by a 25% glucose chaser (250 µL) to prevent hypoglycemia (26). Mice sat for a minimum of 1 week for blood sugar to normalize before infection. Mice were infected, as previously described (26). Briefly, the day before infection mice were anesthetized via isoflurane inhalation, shaved, and treated with Nair on the back. Tail nicks were performed and blood sugar measured via glucometer. The following day, mice were weighed and anesthetized via isoflurane inhalation and injected with lidocaine intra-dermally. Mice were wounded with a 6-mm biopsy punch and inoculated with 1 × 10^7^ CFU GBS. Mice infected with COH1 and COH1 mutants were wrapped with the surgical adhesive Tegaderm for 3 days before adhesive removal and monitoring of animals. Animals were sacrificed 4 days after initial infection. For CJB111, mice were wrapped with Tegaderm for 5 h and sacrificed 2 days post infection. Wound tissues were harvested for further analysis. Tissues were placed into 500 mL of sterile PBS in 2.0-mL conical tubes (Thermo Fisher Scientific) along with 1.0-mm-diameter zirconia/silica beads (BioSpec catalog no. 1107911) and homogenized by bead beating two times for 60 s in a BioSpec mini bead beater. Tissue homogenates were plated on THA and CHROMagar. For polymicrobial infections, mice were inoculated with 5 × 10^6^ GBS and 5 × 10^6^ *E. faecalis*. Wounds were wrapped in Tegaderm for 3 days and mice sacrificed 4 days post infection. For neutrophil depletion experiments, mice were injected with 200 μg of either anti-Ly6G (BioXCell, clone 18A, catalog BE0075-1) or anti-IgG2a isotype control antibody (BioXCell, clone 2A3, catalog BE0089) diluted in buffered reagent diluent (BioXCell, catalog IP0070) the day before infection (51, 78).

### Whole genome sequencing and genome assembly

Genomic DNA from murine wound isolates and the human wound isolate was prepared with the MasterPure Gram Positive DNA Purification Kit (Biosearch Technologies). Illumina short read and Nanopore long read sequencing was performed at SeqCoast. Sequences were aligned to the whole-genome sequence of the parental isolate (COH1 NCBI Reference Sequence: NZ_HG939456.1, or CJB111 GenBank: CP063198.2) and SNPs and SNVs identified using CLC Genomics Workbench. Reads were aligned to the input genomes (COH1 or CJB111) using default parameters in CLC genomics workbench V20 (parameters: Match score 1, mismatch cost 2, linear gap cost, length fraction 0.5, similarity fraction 0.8, non-specific matches were mapped randomLy). The GC content of COH1 is 35.4%, and CJB111 is 35.5%. The sequencing coverage was greater than 135× for all isolates. SNPs were identified using CLC genomic workbench V20 basic variant detection on default parameters using the read alignment to the reference genome (parameters: minimum coverage 10 reads, minimum count 2 reads, minimum frequency 35%). All variants presented in the table were present in greater than 95% of reads mapped, and the average quality of reads >30. The human clinical isolate 216 whole genome was assembled using the Unicyler (v0.5.0) assembly pipeline (79) combining Illumina short reads and Nanopore long reads. The HWI_216 closed and annotated genome (Genbank accession: JBTYLZ000000000) has been deposited into NCBI SRA under BioProject PRJNA1412670. Whole genome sequences of HWI_216 similar isolates were downloaded from Genbank (B37VS: GCF_001349035.1, SG-M426: GCA_034343325.1). Draft genomes were aligned to HWI_216 using the Geneious Mauve plugin (version 1.1.3) MCM alignment algorithm with default parameters (80–82).

### AlphaFold modeling

Structures of the CovS dimer and CovR dimer complexed with the *cylX* promoter region were predicted using AlphaFold3 (41) as implemented on the Protenix Server (83). The structures were visualized using PyMOL (The PyMOL Molecular Graphics System, Version 3.03 Schrödinger, LLC.), and sequence secondary structure diagrams were created using ESPript (84). For prediction of the CovR-DNA complex the 52 base pairs of DNA sequence upstream of COH1 *cylX* gene that corresponded to the region previously shown to be protected from DNAse I digestion by CovR1 was included in the folding prediction.

### Human red blood cell hemolysis

Whole human blood was isolated from healthy human donors (female *n =* 2, male *n* = 2) per the COMIRB protocol number 17-1926. Bacterial cultures were normalized to mid-exponential phase (OD_600_ ~0.4) and re-suspended in PBS. Bacteria, whole blood, and PBS buffer were added to a 1.5 mL Eppendorf tube at a 1:1:1 ratio (200 µL of each component) and incubated at 37 °C for 3.5 h while rotating. After 3.5 h, tubes were centrifuged at 5,500 × *g* for 60 s and 100 µL of supernatant was transferred to a 96-well plate. Erythrocyte lysis was measured as previously described (85) by reading the absorbance at OD_543_.

### HL60 and J774 cytotoxicity

HL60 cells were cultured in RPMI + 10% FBS, differentiated with 1.25% DMSO (Sigma- Aldrich, St. Louis, MO) for 4 days and infected, as previously described (86, 87). J774 cells were cultured in DMEM + 10% FBS, seeded into 96-well plates at 5 × 10^4^ cells/well, and then incubated for 1–2 days at 37 °C with 5% CO_2_. For HL60 and J774 infections, GBS strains were grown to mid-log, and normalized in PBS to 1 × 10^7^ CFU/mL. GBS was added to cells in FBB buffer at an MOI of 10. The plate was incubated for 90 min at 37 °C in an incubator with 5% CO_2_. After infection, 100 µL of supernatant from each well was removed and spun down at 500 × *g* for 1 min to remove cells and debris. Cytotoxicity of cells after infection with GBS strains was determined by LDH release using the CyQUANT LDH Cytotoxicity Assay (Invitrogen by Thermo Fisher C20301, Waltham, MA).

### Nuclease activity assay

The fluorescence resonance energy transfer (FRET)-based nuclease activity assay was performed, as previously described (54, 88, 89). A FRET substrate, ‘‘PrimeTimeTM’’ qPCR probe was purchased from Integrated DNA Technologies consisting of a (15 mer) single-stranded oligonucleotide that is modified at the 5′ end with a Cy3 fluorophore and at the 3′ end with Black Hole Quencher 2 (BHQ2) with the substrate sequence (5′ CCC CGG ATC CAC CCC 3′). Fluorescence measurements were made by mixing 25 µL of FRET substrate, diluted to 2 mM in buffer consisting of 20 mM Tris pH 8.0 and 10 mM CaCl_2_, with 25 µL of supernatant from GBS grown overnight at 37°C. Fluorescence measurements were taken by measuring the rate of fluorescence change (excitation 552 nm/emission 580 nm) at 30°C in a Tecan Infinity 200 M plate reader.

### *In vitro* growth and competition assays

GBS strains were grown in THB overnight for 15 h in a static incubator at 37°C. The following morning, 1 mL of culture was spun down in a centrifuge at 13,000 rpm and the pellet resuspended in sterile PBS. Cultures in PBS were diluted 1:200 into fresh media of either THB in a 96 well plate in technical triplicate. The OD_600_ of plates was recorded every hour to determine growth.

*In vitro* competition between GBS and *E. faecalis* was conducted as previously described with some modifications (90). GBS strains and *E. faecalis* OG1RF were grown in THB overnight for 15 h in static (GBS) or shaking (*E. faecalis*) conditions at 37°C. Overnight cultures were pelleted at 13,000 rpm and resuspended in fresh THB before normalizing to an OD of 0.4. Normalized cultures were then diluted to ~5 × 10^6^ CFU/mL in THB. Experimental cultures were prepared by mixing 1 mL of prepared COH1 or 90-CovR_T218A_ with 1 mL of prepared OG1RF. Cultures were vortexed to mix and statically incubated at 37°C. Every 2 h, 25-µL aliquots from cultures were serially diluted in PBS before plating on CHROMagar (GBS) and THA with 50 µg/mL rifampin and 25 µg/mL fusidic acid (OG1RF) to enumerate CFU. Competitive index was calculated as the experimental ratio of GBS:OG1RF versus the input ratio. All experiments were conducted in technical triplicate.

### Statistical analyses

All statistics were performed using GraphPad Prism software V10. All relevant statistical analyses are indicated in the figure legends.

## SUPPLEMENTAL MATERIAL

Supplemental material iai.00099-26-s0001.pdf Fig. S1 to S6; Table S1.

ASM does not own the copyrights to Supplemental Material that may be linked to, or accessed through, an article. The authors have granted ASM a non-exclusive, world-wide license to publish the Supplemental Material files. Please contact the corresponding author directly for reuse.
