# Supplementary material for: The diabetic wound microenvironment drives emergence and maintenance of CovRS variants in group B Streptococcus
Source: Infect Immun. 2026 Jun 15;94(7):e00099-26. doi: 10.1128/iai.00099-26 (PMC13367057; doi:10.1128/iai.00099-26)
Supplement: Supplemental material — Fig. S1 to S6; Table S1. [file iai.00099-26-s0001.pdf]

Supplemental Figures

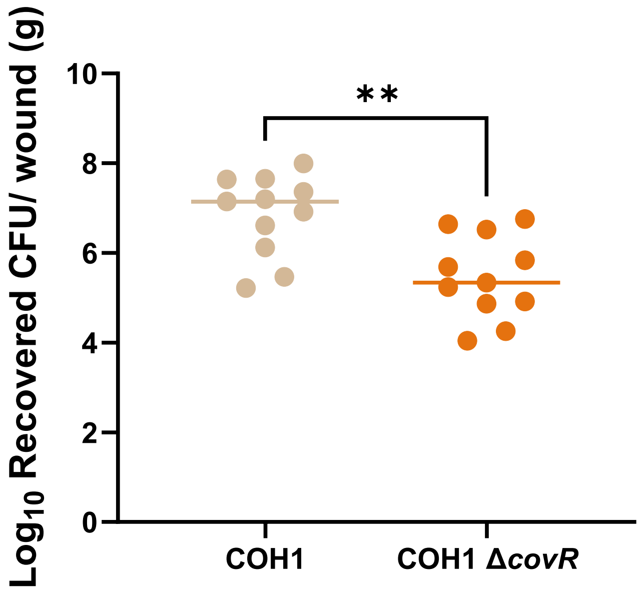

Supplemental Figure 1. A *covR* mutant has reduced fitness in a healthy host. CFU of GBS recovered from wound homogenate. Line at median graph. Significance determined via Mann-Whitney U-test; ***p*<.01.

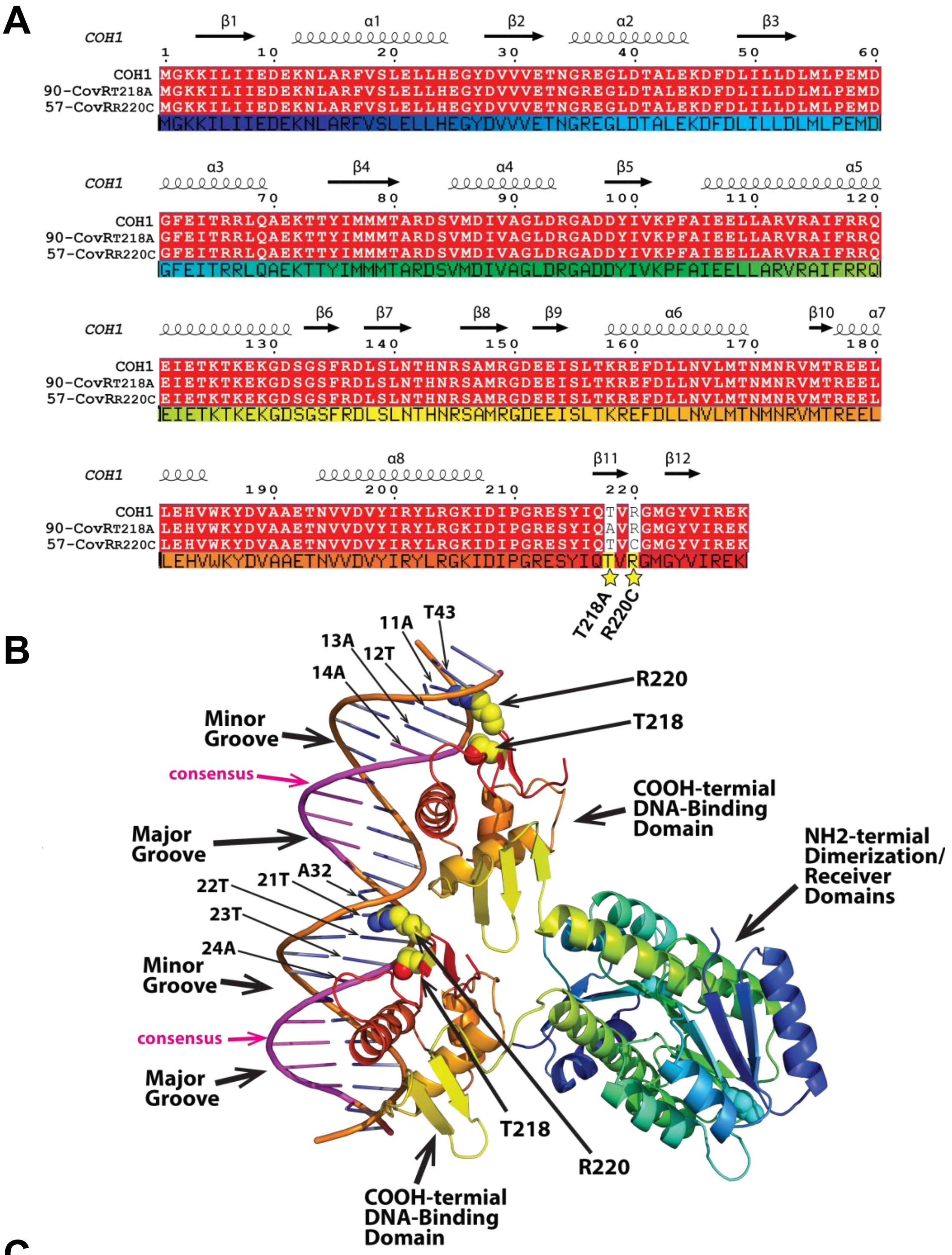

C

```
                  1111111111222222222233333333334444444444555 5
base#        1234567890123456789012345678901234567890123456789012
consensus               ATTARAQ      ATTARAG   R=A/G and Q=A/T
sense        TTTTAACATAATAATGATATTTTAATTAGAGTGTGAATTTATAATTAATTTT
antisense    AAAATTGTATTATTACTATAAAATTAATCTCACACTTAAATATTAATTAAAA
base#        5554444444444333333333322222222221111111111
             2109876543210987654321098765432109876543210987654321
```

Supplemental Figure 2. CovR Modeling of 90-CovR$_{T218A}$ and 57-CovR$_{R220C}$. (A) Primary sequence of CovR in COH1, 90-CovR$_{T218A}$ and 57-CovR$_{R220C}$. Mutation sites indicated by yellow stars (B) Ribbon diagram with colors corresponding to primary sequence. (C) Predicted docking sequence of the CovR dimer onto the *cylX* promoter. CovR consensus binding sequence highlighted in magenta.

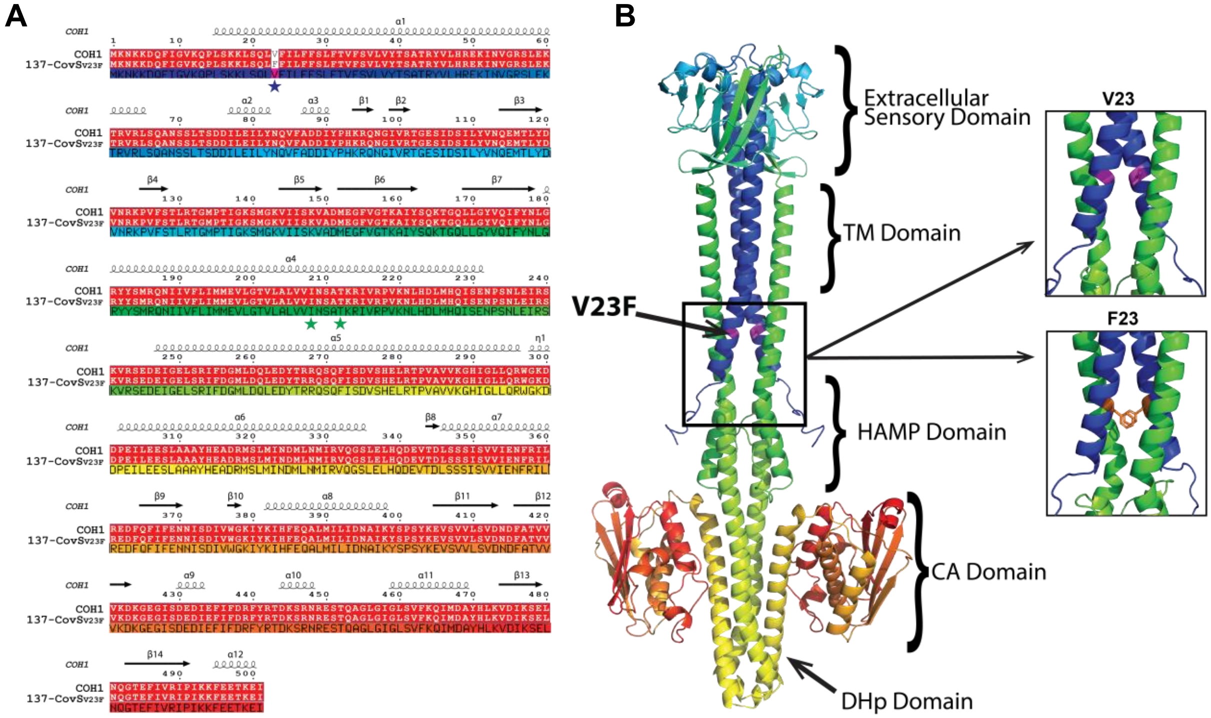

Supplemental Figure 3. CovS Modeling of 137-CovS$_{V23F}$. (A) Primary sequence of CovS in COH1 and 137-CovS$_{V23F}$. Mutation site indicated by blue star. The α8 helix interaction sites from the other subunit are indicated by the green stars (B) Ribbon diagram with colors corresponding to primary sequence.

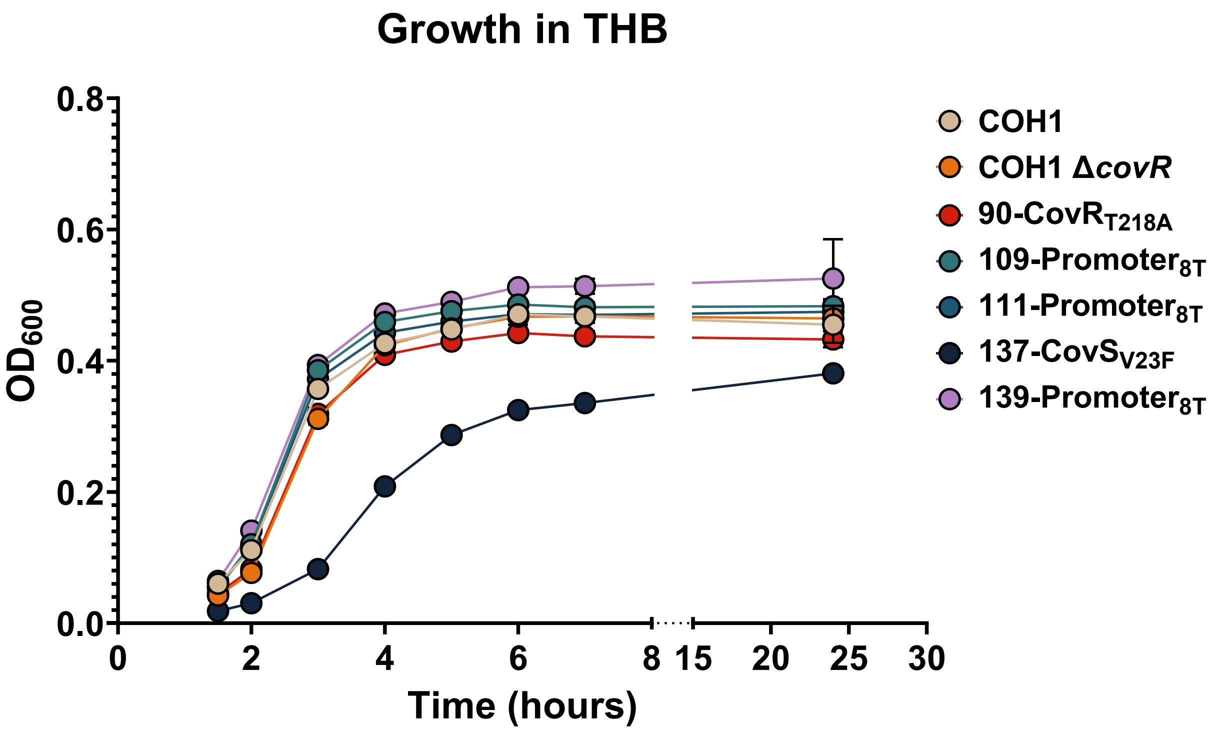

Supplemental Figure 4. Growth Assessment of Murine Wound Isolates. (A) Growth curve of MWIs in THB. Error bars demonstrate geometric mean with error in technical triplicate.

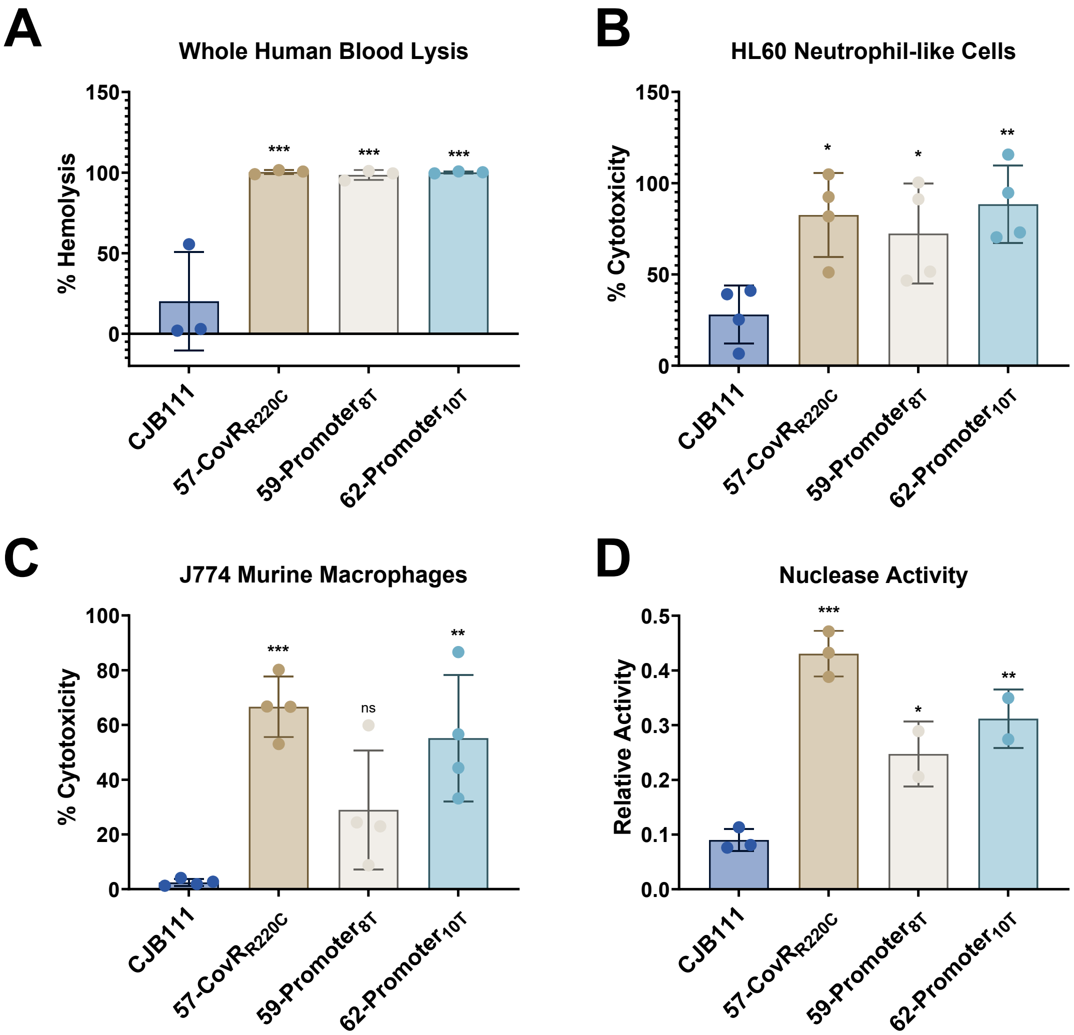

Supplemental Figure 5. Hyper-Pigmented Isolates Exhibit Enhanced Virulence Output. (A) Hemolysis of human red blood cells after 3.5 h incubation. (B) Cytotoxicity via LDH release of HL60s of (C) J774s after 1.5 h of incubation at MOI of 1. (D) Relative nuclease activity. All error bars represent means with SD. Panels A and D performed in biological triplicate or duplicate. Panels B and C performed in biological quadruplicate. Significance determined via one-way ANOVA with Dunnett's multiple comparisons against WT as a control group; *p<.05, **p<.01, ***p<.001, ****<.0001, ns= not significant.

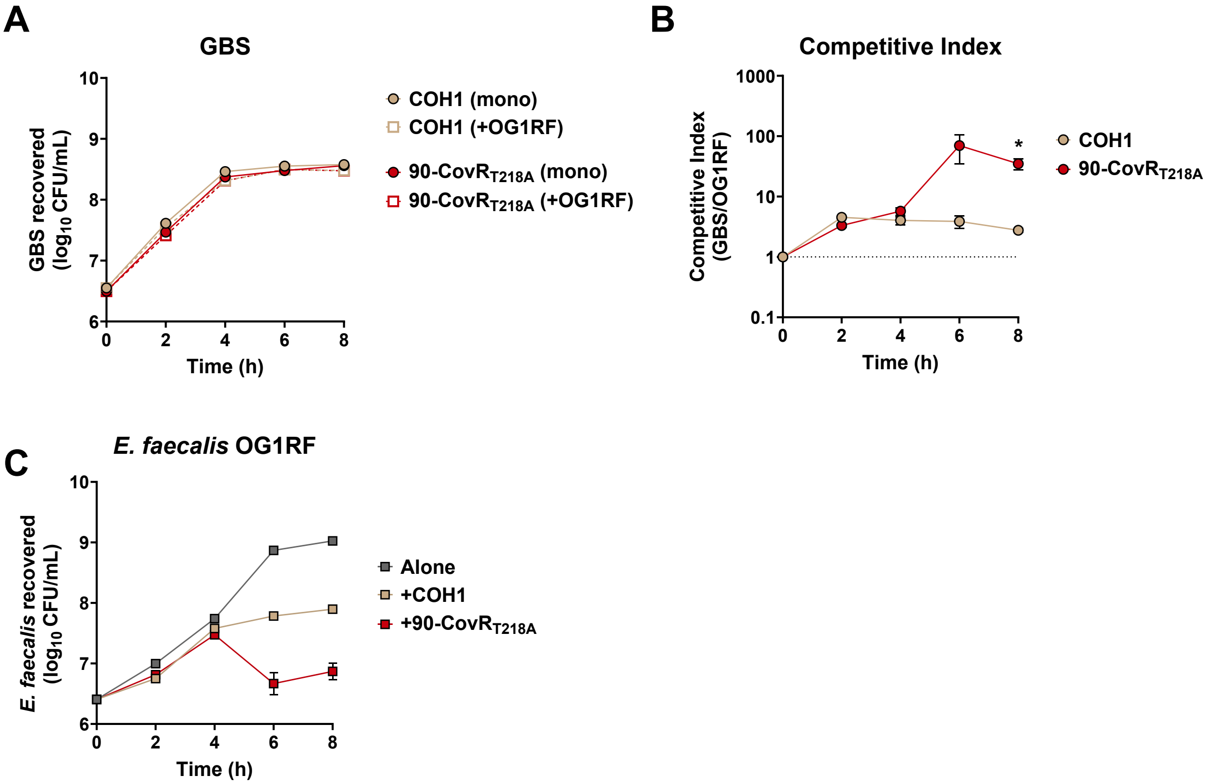

Supplemental Figure 6. 90-CovR$_{T218A}$ outcompetes *E. faecalis in vitro*. (A) Growth of COH1 and 90-CovR$_{T218A}$ with and without OG1RF. (B) Competitive index of COH1 and 90-CovR$_{T218A}$ /OG1RF. (C) *E. faecalis* CFU in the presence of COH1 and 90-CovR$_{T218A}$. Significance determined via Multiple lognormal t-tests with Holm-Šídák correction for multiple comparisons *p<0.05.

Supplemental Table 1. Bacterial strains used in this study

Bacterial Strain	Source	Identifier
GBS COH1 WT	(1)	COH1
GBS COH1 Δ*covR*	(2)	COH1 ΔcovR
GBS 90-CovR$_{T218A}$	This work	90-CovR$_{T218A}$
GBS 109-Promoter$_{8T}$	This work	109-Promoter$_{8T}$
GBS 111-Promoter$_{8T}$	This work	111-Promoter$_{8T}$
GBS 137-CovS$_{V23F}$	This work	137-CovS$_{V23F}$
GBS 139-Promoter$_{8T}$	This work	139-Promoter$_{8T}$
GBS CJB111	(3)	CJB111
GBS 57-CovR$_{R220C}$	This work	57-CovR$_{R220C}$
GBS 59-Promoter$_{8T}$	This work	59-Promoter$_{8T}$
GBS 62-Promoter$_{10T}$	This work	62-Promoter$_{10T}$
GBS HWI_216	(4)	HWI_216
E. faecalis OG1RF	(5)	OG1RF

Supplemental References

1. Wilson CB, Weaver WM. 1985. Comparative Susceptibility of Group B Streptococci and Staphylococcus aureus to Killing by Oxygen Metabolites. J Infect Dis 152:323–329.

2. Patras KA, Wang NY, Fletcher EM, Cavaco CK, Jimenez A, Garg M, Fierer J, Sheen TR, Rajagopal L, Doran KS. 2013. Group B Streptococcus CovR regulation modulates host immune signalling pathways to promote vaginal colonization. Cell Microbiol 15:1154–1167.

3. Flores AR, Galloway-Peña J, Sahasrabhojane P, Saldaña M, Yao H, Su X, Ajami NJ, Holder ME, Petrosino JF, Thompson E, Ros IMY, Rosini R, Grandi G, Horstmann N, Teatero S, McGeer A, Fittipaldi N, Rappuoli R, Baker CJ, Shelburne SA. 2015. Sequence type 1 group B Streptococcus, an emerging cause of invasive disease in adults, evolves by small genetic changes. Proc Natl Acad Sci U S A 112:6431–6436.

4. Keogh RA, Huyvaert S, Moore GD, Horswill AR, Doran KS. 2024. Virulence characteristics of Gram-positive bacteria isolated from diabetic foot ulcers. FEMS Microbes 5.

5. Bourgogne A, Garsin DA, Qin X, Singh K V., Sillanpaa J, Yerrapragada S, Ding Y, Dugan-Rocha S, Buhay C, Shen H, Chen G, Williams G, Muzny D, Maadani A, Fox KA, Gioia J, Chen L, Shang Y, Arias CA, Nallapareddy SR, Zhao M, Prakash VP, Chowdhury S, Jiang H, Gibbs RA, Murray BE, Highlander SK, Weinstock GM. 2008. Large scale variation in Enterococcus faecalis illustrated by the genome analysis of strain OG1RF. Genome Biol 9.
